# Effects of Inclusion of Different Doses of *Persicaria odorata* Leaf Meal (POLM) in Broiler Chicken Feed on Biochemical and Haematological Blood Indicators and Liver Histomorphological Changes

**DOI:** 10.3390/ani10071209

**Published:** 2020-07-16

**Authors:** Muhammad Abdul Basit, Arifah Abdul Kadir, Teck Chwen Loh, Saleha Abdul Aziz, Annas Salleh, Ubedullah Kaka, Sherifat Banke Idris

**Affiliations:** 1 Department of Preclinical Sciences, Faculty of Veterinary Medicine, Universiti Putra Malaysia, UPM, Serdang 43400, Selangor, Malaysia; bankidris67@gmail.com; 2 Department of Biosciences, Faculty of Veterinary Sciences, Bahauddin Zakariya University, Multan 60000, Punjab, Pakistan; 3 Department of Animal Sciences, Faculty of Agriculture, Universiti Putra Malaysia, UPM, Serdang 43400, Selangor, Malaysia; tcloh@upm.edu.my; 4 Department of Veterinary Pathology & Microbiology, Faculty of Veterinary Medicine, Universiti Putra Malaysia, UPM, Serdang 43400, Selangor, Malaysia; saleha@upm.edu.my; 5 Department of Veterinary Laboratory Diagnostics, Faculty of Veterinary Medicine, Universiti Putra Malaysia, UPM, Serdang 43400, Selangor, Malaysia; annas@upm.edu.my; 6 Department of Companion Animal Medicine and Surgery, Faculty of Veterinary Medicine, Universiti Putra Malaysia, UPM, Serdang 43400, Selangor, Malaysia; dr_ubedkaka@hotmail.com; 7 Department of Veterinary Pharmacology and Toxicology, Faculty of Veterinary Medicine, Usmanu Danfodiyo University, 2346 Skoto, Nigeria

**Keywords:** broiler chicken, feed additive, blood haematology, phytobiotics, serum biochemistry

## Abstract

**Simple Summary:**

The frequent use of antimicrobial growth promoters (AGPs) in poultry feed leads to antimicrobial resistance, resulting in a ban on their subtherapeutic use in food-producing animals. In this context, there is a dire need to find safe and potential alternatives. Recently, phytobiotics, especially herbs, have gained attention and have been studied extensively for their possible use as alternative poultry feed additives. *Persicaria odorata* is a herb (phytobiotic) that is reported to possess antioxidant, antimicrobial, immunomodulatory, and hepatoprotective properties. This study is the first of its kind to assess the effects of different doses of supplementation of *Persicaria odorata* leaf meal (POLM) on haematological blood indicators, serum biochemistry, organ parameters, and histomorphology of the liver in broiler chickens. The results revealed that the dietary supplementation of POLM enhanced the growth performance, positively improved the haematological indices and serum biochemistry profile with no deleterious effects on internal organs, and ameliorated the histomorphology of the liver, even at dietary supplementation of 8 g/kg. Thus, POLM would be safe at an inclusion rate of 8 g/kg as an alternative phytogenic feed additive in broiler chickens.

**Abstract:**

This research was conducted to estimate the effects of *Persicaria odorata* leaf meal (POLM) on haematological indices, serum biochemical attributes, and internal organs parameters, including histomorphological features of the liver, in broiler chickens. A total of 120 one-day-old male broiler chicks (Cobb-500) were randomly allocated into four experimental groups. The dietary treatments were basal diet (BD), which served as the control (C), along with BD + 2 g/kg POLM (Po2), BD + 4 g/kg POLM (Po4), BD + 8 g/kg POLM (Po8), which were the supplemented groups. The body weight gain (BWG) showed a linear increase and feed conversion ratio (FCR) showed a linear decrease with increasing POLM dosage at day 42 (*p* ˂ 0.05) and for the overall growth performance period (*p* ˂ 0.01). On day 21 and day 42, the values of red blood cells (RBCs), white blood cells (WBCs), haemoglobin (Hb), and packed cell volume (PCV) showed linear increases (*p* ˂0.05) as the dosage of POLM increased in the diet. On day 21, dietary supplementation of POLM linearly decreased (*p* ˂ 0.05) the serum activity of alkaline phosphatase (ALP), aspartate aminotransaminase (AST), alanine aminotransaminase (ALT), and serum levels of urea and creatinine. On the other hand, serum levels of total protein (TP), albumin, and globulin showed a linear increase (*p* ˂ 0.05) as the POLM dosage increased. On day 42, the serum activity of AST and ALT and serum levels of glucose, cholesterol, triglycerides, urea, and creatinine showed linear decreases (*p* ˂ 0.05) with increased levels of POLM in the diet. However, POLM supplementation linearly increased (*p* ˂ 0.05) the serum levels of TP and globulin. Dietary inclusion of POLM did not influence the organ parameters and showed no adverse effects on the liver histomorphology. In conclusion, supplementation of POLM increased the growth performance, improving haematological indices and serum biochemistry profiles of broiler chickens without any deleterious effects on the liver histomorphology. The results of the present study provide evidence that POLM can be safely used at a dose rate of 8 g/kg of feed as an alternative to conventional antimicrobial growth promoters (AGPs).

## 1. Introduction

The ban against in-feed inclusion of antimicrobial growth promoters (AGPs) has increased the momentum in research to find potential alternatives [1,2]. An increasing interest has been seen in the study of phytobiotics as alternatives to AGPs. Among phytobiotics, herbs are of particular significance because of their secondary bioactive metabolites, such as flavonoids, which are potent antioxidants, thus helping to prevent oxidative stress and reduce the risk of developing chronic diseases [3,4]. Additionally, they are anti-inflammatory, immunomodulatory [5,6], antimicrobial, anthelmintic, [3,4,7], detoxifying, and digestion-stimulating substances [8].

Herbs have shown positive effects on the performance and biological health of broiler chickens [9,10], can improve haematological blood indicators and serum biochemical attributes [11,12,13], and have also been reported to regulate the kidney and liver functions [14,15]. Among such herbs, *Persicaria odorata*, of the family Polygonaceae, is of important significance. *P. odorata* is a perennial herb that can grow up to 1.0 m in lowlands and 1.5 m in hilly areas. This plant possesses long and lanceolate leaves measuring 0.5–2.0 cm in width and 5–7 cm in length [16,17], and is used traditionally and regularly in Southeast Asian cuisine. *P. odorata* has many common names, such as Vietnamese cilantro and Vietnamese mint. In Malaysia, Indonesia, Singapore, and Brunei, it is called “*daun laksa*” or “*daun kesum*” [16]. The dried grounded leaves of *P. odorata* contain 3.5% crude protein, 0.83% crude fat, 10.66% crude fiber, and 1.83% ash [18]. It is a powerful antioxidant [17] that contains essential oils [19] and flavonoids [20]. Among these flavonoids, myricetin, quercetin, gallic acid, and coumaric acid are essential bioactive compounds [21]. Its high polyphenolic content, quercetin and myricetin, has been suggested to be responsible for its antioxidant activity [22,23], which can inhibit lipid peroxidation [24]. Moreover, previous studies have shown that *P. odorata* was non-toxic in murine model [25,26].

Phytobiotics are assumed to be natural, safe, and residue-free substances, which may have mildly toxic effects compared to commonly used synthetic AGPs [27]. Literature is scarce about the safe use of herbal plants and their optimal dosage; thus, there is a dire need to estimate the appropriate dosage and the possible side effects of natural feed additives, which might be used as safe alternative growth promoters in poultry production. The present study aims to estimate the effects of different doses of supplementation of POLM on the growth performance, haematological blood indicators, serum biochemical attributes, histomorphology of the liver, and internal organ parameters in broiler chickens. 

## 2. Materials and Methods

### 2.1. Source and Preparation Method for Diets

Fresh samples of *P. odorata* were obtained from the Universiti Putra Malaysia herbal farm. The obtained samples were authenticated from the Biodiversity Unit, Institute of Biosciences, Universiti Putra Malaysia, and deposited with voucher no. SK3296/18 for future reference. For sample preparation, fresh leaves of *P. odorata* were dried using an oven set to 50 °C for 72 h and milled to a fine powder. The obtained sample (fine powder) was stored at 4 °C until further use.

### 2.2. Experimental Birds and Diets

The experimental procedures and animal handling were approved by the institutional animal care and use committee, Universiti Putra Malaysia (UPM/IACUC/AUP-R033/2018). A total of 120 one-day-old male broiler chicks (Cobb500) were procured from a local hatchery. Upon arrival, the birds were wing-tagged and allocated randomly into 4 treatment groups with 5 replicates of 6 birds each. The birds were raised in cages with wire meshed floor (length 120 cm × width 120 cm × height 45 cm), which were placed in a conventional open-sided shed. The birds in all replicates were reared under the same environmental and management conditions. The cyclic temperature in the house ranged between a maximum of 34 °C and a minimum of 24 °C, while the humidity ranged between a maximum of 91% and a minimum of 65%. Commencing from day one, birds had free access to water and feed, while the lighting was continuous. The basal diet without any premixing of anticoccidial, antimicrobial, and antioxidant drugs or feed enzymes was procured from the feed supplier and processed into experimental diets at a feed mixing facility at the Universiti Putra Malaysia. The current study consisted of four dietary treatments, which were given for 42 days (starter period = 1 to 21days, finisher period = 22 to 42 days). The dietary treatments were the basal diet (BD), which served as the control (C); and BD + 2 g/kg POLM (Po2), BD + 4 g/kg POLM (Po4), and BD + 8 g/kg POLM (Po8), which were the treatment groups. The experimental diets were formulated in the composition that meets or exceed NRC [28] recommendations (Table 1). The experimental broiler chickens were vaccinated with Newcastle disease (ND) and infectious bronchitis (IB) vaccines at day 4 and day 21, respectively; and vaccinated with the infectious bursal disease (IBD) vaccine on day 7 via an intra-ocular route. 

### 2.3. Growth Performance Measurement

The initial body weights of the birds were recorded on day 1, followed by weekly recording of live body weight (BW) and feed intake (FI) throughout the entire experiment. The body weight gain (BWG) and feed conversion ratio (FCR) were calculated. Mortality was recorded whenever it occurred.

### 2.4. Sample Collection

On day 21 and day 42, two broiler chickens were randomly selected per replicate (*n* = 10) per experimental group. From each bird, the blood samples were collected via brachial (wing) vein to check haematological blood indicators and serum biochemical indices.

### 2.5. Analysis of Haematological Blood Indicators

For haematological blood indicator analyses, the blood samples were collected in the K_3_ EDTA tubes (BD Vacutainer^®^, Franklin Lakes, NJ, USA). Blood haematology parameters, including red blood cell (RBCs) and white blood cell (WBCs) counts, haemoglobin (Hb), packed cell volume (PCV), mean corpuscular volume (MCV), mean corpuscular haemoglobin (MCH), and mean corpuscular haemoglobin concentrations (MCHC) were measured within 2 h post blood collection using a haematology analyser (ABC Vet^®^, ABX Diagnostics, Montpellier, France).

### 2.6. Serum Biochemical Indices

For serum biochemical analyses, blood samples were collected in a plain tube (BD Vacutainer^®^, Franklin Lakes, NJ, USA) and subjected to centrifuge at 3000×*g* for 15 min to obtain serum, which was stored at −20 °C until further analyses. The serum biochemical indices, including aspartate aminotransaminase (AST), alanine aminotransaminase (ALT), alkaline phosphatase (ALP), urea, creatinine, triglycerides, cholesterol, glucose, total protein (TP), albumin, and globulin; and serum electrolytes, including sodium (Na), chloride (Cl), and potassium (K), were measured with commercial kits (Roche “Basal” Diagnostica, Rotkreuz, Switzerland) using an autochemistry analyser (Bio Lis 24i Chemistry Analyser, Tokyo, Japan).

### 2.7. Determination of Relative Internal Organ Weights

On day 42, two birds per replicate (*n* = 10) from each experimental group were selected randomly and slaughtered according to the procedure designated in Malaysian Standard (MS) 1500: 2009 [29]. The following equation was used to calculate the dressing percentage (DP):

DP = (eviscerated carcass weight/live weight) × 100(1)

The weights of the gizzard, heart, liver, kidney, spleen, pancreas, and bursa of Fabricius were taken and expressed as percentages (%) of the live body weight.

### 2.8. Liver Histomorphology Assessment

The liver samples were collected from birds slaughtered to determine internal organ parameters. For histomorphological studies, the liver tissues were kept for 48 h in 10% buffered formalin and subjected to a series of dehydration cycles in an automated tissue processor (Leica ASP 3000, Wetzlar, Germany). The liver tissues were embedded using a paraffin embedding system (Leica RM 2155, Wetzlar, Germany). Tissue sections up to 4–5 µm in size were obtained using a microtome (Leica Jung Multicut 2045, Wetzlar, Germany) and stained using haematoxylin and eosin staining. For the histomorphology, the tissues were examined under a light microscope (Leica DM LB2, Wetzlar, Germany). 

### 2.9. Data Analyses

The data analyses were carried out with SAS 9.4 [30] (SAS Institute Inc., Cary, NC, USA) by one-way ANOVA using the general linear model procedure. Group differences were compared by Duncan’s multiple range test. The effects of POLM supplementation at different doses were measured using an orthogonal polynomial contrast test for linear and quadratic effects. The differences were considered as significant at *p* < 0.05. 

## 3. Results

### 3.1. Growth Performance

The growth performance of broiler chickens is shown in Table 2. On day 21, compared to the control BWG was significantly increased (*p* ˂ 0.05) in the Po8 group; however, FI and FCR were not affected (*p* > 0.05) by dietary supplementation of POLM. On day 42, compared to the control, BWG was significantly increased (*p* ˂ 0.05) in POLM-supplemented groups (Po2, Po4, and Po8). Furthermore, FCR was decreased (*p* < 0.05) in POLM-supplemented diets compared to the control group. Additionally, FI was not affected (*p* > 0.05) by dietary supplementation of POLM. Regarding the overall growth performance of broiler chickens (days 1–42), the maximal increase (*p* ˂ 0.05) for the BWG and the lowest (*p* ˂ 0.05) FCR were seen in dietary group Po8 compared to the control group. In addition, the BWG showed a linear increase (*p* ˂ 0.05) and FCR showed a linear decrease (*p* ˂ 0.05) with increasing POLM dosage on day 42 (*p* ˂ 0.05) and for the overall growth performance period.

### 3.2. Haematological Blood Indicators

Data in Table 3 and Table 4 illustrate the impacts of different doses of POLM on the haematological blood indicators of broilers on day 21 and day 42, respectively. On day 21, compared to the control, RBC counts were improved (*p* < 0.05) in the Po8 group. On the other hand, PCV was significantly increased in the POLM-supplemented groups (Po2, Po4, and Po8) compared with the control group. The Hb concentration was higher (*p* < 0.05) in the Po4 group and the maximum value (*p* < 0.05) was recorded in the Po8 group compared to the control group. Furthermore, the WBC counts were increased (*p* < 0.05) in dietary group Po8 relative to the control group. However, MCV, MCH, and MCHC were not affected (*p* > 0.05) by the dietary supplementation of POLM. In addition, POLM supplementation linearly increased RBC (*p* = 0.048), Hb (*p* = 0.048), and WBC counts (*p* = 0.0001) with increasing POLM dosage.

On day 42, compared with the control group, RBC, WBC, PVC, and Hb counts increased (*p* < 0.05) in the Po8 group. On the other hand, POLM supplementation did not influence (*p* > 0.05) MCV, MCH, or MCHC values in experimental broiler chickens. In addition, POLM supplementation linearly increased RBC (*p* = 0.046), Hb (*p* = 0.047), MCV (*p* = 0.016), MCHC (*p* = 0.039), and WBC counts (*p* = 0.0001) with increasing POLM dosage.

### 3.3. Serum Biochemistry

Data in Table 5 and Table 6 show the influences of different doses of POLM on the serum biochemistry of broilers on day 21 and day 42, respectively. On day 21, compared to the control group, the AST activity in the serum was significantly decreased (*p* < 0.05) with increasing levels of POLM supplementation. However, the serum activity of ALT was decreased in Po8 group compared with the control group. On the other hand, the activity of ALP in the serum was not affected (*p* > 0.05) by dietary supplementation of POLM. The serum TP level was increased (*p* < 0.05) in dietary group Po8 compared to the control group. Furthermore, the serum levels of albumin and globulin (except Po2) were increased (*p* < 0.05) in POLM-supplemented groups compared to the control group. In contrast with the control group, except for Po2, the serum levels of urea and creatinine were decreased (*p* < 0.05) in POLM-supplemented groups. On the other hand, supplementation of POLM did not influence (*p* > 0.05) serum levels of glucose, cholesterol, triglycerides, K, or Cl in experimental broiler chickens. In addition, there was a linear decrease in the serum activity levels of ALP (*p* = 0.048) and AST (*p* = 0.000) and the serum levels of urea (*p* = 0.044) and creatinine (*p* = 0.000) as the POLM dosage increased. However, a linear and quadratic decrease in the serum activity of ALT (*p* = 0.000) was observed with increasing POLM dosage. The POLM supplementation linearly increased the serum levels of TP (*p* = 0.000), albumin (*p* = 0.022), and globulin (*p* = 0.000) with increasing supplementation levels in the diet.

On day 42, the activity of AST and ALT in the serum was decreased (*p* < 0.05) with increasing POLM supplementation. However, the serum activity of ALP was not influenced by (*p* > 0.05) POLM supplementation in experimental broilers. The serum level of TP was increased (*p* < 0.05) in POLM supplementation groups compared to the control group. The serum levels of albumin in the Po8 group and globulin in Po4 and Po8 groups were significantly increased (*p* < 0.05) compared with the control group. Serum levels of cholesterol, triglycerides, and urea were decreased (*p* < 0.05) with increasing POLM supplementation levels, in comparison with the control group. Birds in Po8 and Po4 groups showed decreased serum creatinine levels compared with the Po2 and control groups. However, POLM supplementation did not vary (*p* > 0.05) the serum level of Na, Cl, or glucose in the experimental birds. In addition, there was a linear decrease in serum levels of AST (*p* = 0.003), glucose (*p* = 0.002), cholesterol (*p* = 0.000), and triglycerides (*p* = 0.000) as the POLM supplementation increased. Furthermore, linear and quadratic decreases in serum levels of ALT (*p* = 0.000; 0.004), urea (*p* = 0.000; 0.03), and creatinine (0.000; 0.022) were observed as the POLM supplementation levels increased. Moreover, the serum levels of TP (*p* = 0.001) and globulin (*p* = 0.006) showed linear increases with increased supplementation of POLM.

### 3.4. Dressing Percentage and Relative Internal Organs Weights

The dietary supplementation of POLM did not influence (*p* > 0.05) the dressing percentages or relative internal organs weights across experimental broiler chickens (Table 7). Moreover, there were linear increases in the dressing percentage (*p* = 0.040) and relative spleen weight (*p* = 0.000) with increasing levels of POLM.

### 3.5. Morphological Analyses of Liver

**Figure 1:**
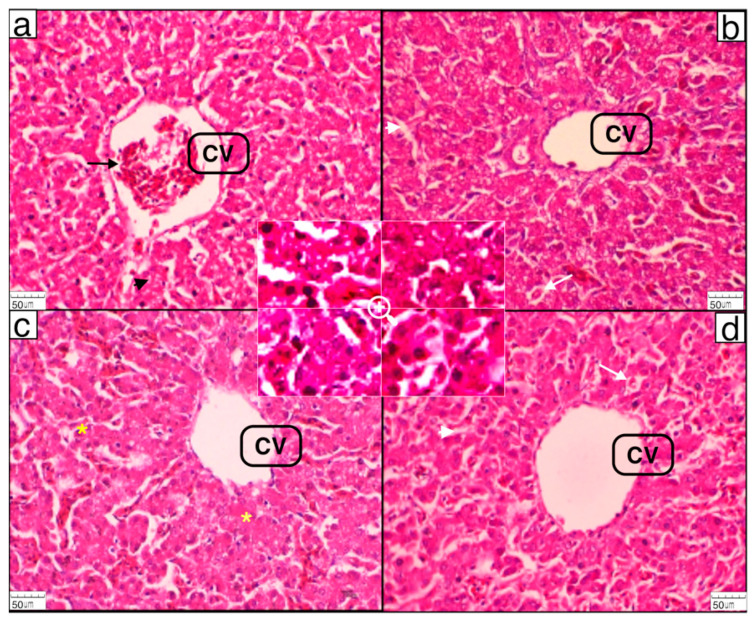
Photomicrograph image. (**a**) Liver lobule section of the control group, showing partial congestion (black arrow) in the central vein (CV) and vacuolar degeneration of the hepatocytes (black arrowhead). (**b–d**) Liver lobule sections of the Po2, Po4, and Po8 groups, showing central veins with intact endothelia, RBCs within sinusoids (white arrow), and radiating sinusoidal spaces (white arrowhead); there is no evidence of the infiltration of inflammatory cells in the liver parenchyma. (**c**) Hepatocytes showing normal architecture (asterisk)**.** The magnified area in the center of the image shows histomorphological features of hepatocytes, where the hepatocytes in the Po8 group (**d**) have the clearest and healthiest architecture compared to the other groups (H&E:Haematoxylin and Eosin; 400X).

Figure 1a–d show the histomorphologies of the liver sections of the experimental birds. The histomorphological examination of the liver section of the control group showed congestion of the central vein (CV), with loosening of the endothelium and vacuolar degeneration of the hepatocytes (Figure 1a). The liver lobule sections of the POLM-supplemented groups (Po2, Po4, and Po8) showed normal architecture of the hepatic lobules and central veins with intact endothelia and hepatic sinusoids. Furthermore, multifocal areas of the RBCs were seen in sinusoidal capillaries, without any infiltrative evidence of inflammatory cells within the liver parenchyma (Figure 1b–d). A gradual histomorphological improvement in the normalcy level of the hepatocytes was noticed with increasing POLM supplementation levels. The hepatocyte architecture was clearer and showed no vacuolation or degenerative changes in the Po8 group compared to Po2 and Po4, where low levels of vacuolation and fatty changes were noticed within hepatocytes in a centrally magnified area of the hepatic lobule sections. 

## 4. Discussion

### 4.1. Growth Performance

Supplementation of POLM enhanced the growth performance of broiler chickens. In this study, compared to the control group, BWG was significantly increased (*p* ˂ 0.05) in the Po8 group on day 21; however, FI and FCR were not affected (*p* > 0.05) by dietary supplementation of POLM. Aroche et al. [31] reported that the dietary inclusion of phytobiotics in the form of 0.5 % mixed powder of *M. citrifolia*, *P. guajava*, and *A. occidentale* improved the feed efficiency, which resulted in increased BWG. The *P. guajava and M. citrifolia* contain flavonoids and possess antioxidant and antimicrobial properties [32]. The flavonoid contents of these phytobiotics are assumed to increase the growth performance in supplemented broiler chickens [33,34]. The secondary metabolites of herbs such as alkaloids, tannins, and flavonoids positively influence the birds’ health, as they possess antimicrobial, anti-inflammatory, and antioxidant properties [35]. Thus, the inclusion of phytobiotics as a dietary supplement help to improve the growth in chickens [36,37,38].

On day 42, compared to the control group, BWG was increased (*p* ˂ 0.05) and FCR was significantly decreased in POLM-supplemented groups (Po2, Po4, and Po8). Additionally, The BWG showed a linear increase and FCR showed a linear decrease (*p* ˂ 0.05) with increasing POLM dosage. Supplementation of POLM resulted in enhanced BWG in broiler chickens, which may have been due to the flavonoids and the secondary bioactive compound quercetin, which has a primary role and was successfully quantified from POLM. Quercetin is a flavone that should improve growth in birds by upregulating growth hormone, triggering the hepatic growth hormone receptor; this stimulus increases the concentration of insulin-like growth factor-1 [39]. Kim et al. [40] reported increased broiler growth by supplementing quercetin in broilers. Quercetin can limit the effects of oxidative stress [41] and pro-inflammatory cytokines such as TNF-α, interleukin, and cyclooxygenase-2 [42], thus modulating the gut environment to better utilise the nutrients, improving the growth in birds. 

The overall growth performance (1–42 days) of broiler chickens showed that compared to the control group, BWG was maximumly increased (*p* ˂ 0.05) and FCR was significantly decreased (*p* ˂ 0.05) in the Po8 dietary group. In addition, the BWG showed a linear increase (*p* ˂ 0.01) and FCR showed a linear decrease (*p* ˂ 0.01) with increasing POLM dosage. Salami et al. [43] identified that the incorporation of medicinal herbs as feed additives in the broiler chickens’ diets improved the FCR in the last growth phase of the birds. Other studies also suggested the roles of flavonoids in the growth performance of broiler chickens [34,44]. The present study results are in agreement with Mpofu et al. [45], where inclusion of *L. javanica* at the rate of 5 g/kg in broiler chickens’ diets had a positive impact on overall growth. In another study, Paraskeuas et al. [36] reported that inclusion of phytobiotics such as eugenol, menthol, and anethol at the rate of 100 to 150 mg/kg in feed improved the nutrient digestibility, thus enhancing the growth measures in broiler chickens.

Conclusively, the supplementation of POLM in broiler chickens showed a positive effect on growth performance, hence effectively increasing the BWG and feed efficiency with decreased FCR. These findings are in agreement with the positive results of the previous studies, where in-feed phytobiotics were tested in broiler chickens [31,45,46]. Thus, the present results show that POLM effectively enhanced the growth performance of broiler chickens, even at the supplementation rate of 8 g/kg.

### 4.2. Haematological Blood Indicators

Haematology blood tests of experimental animals are very significant when evaluating the toxic effects of a supplemented compound or plant extract. Haematology blood tests are also tools that can be used to determine the physiological and pathological statuses of the organisms [47]. The haematological blood indicators in this study were found to be within normal ranges [48]. The normal blood haematology values in this study indicated the adequacy of nutrients and better immune status of the broiler chickens supplemented with POLM.

The current study findings indicated significant increases in RBC and WBC counts, and in Hb and PCV values. These outcomes are comparable with the results of Reis et al. [49], who indicated that inclusion of phytobiotics such as cinnamic aldehyde, thymol, and carvacrol in broiler chickens significantly increased erythrocyte counts and haemoglobin in comparison with the control. Similar findings in another study were reported by Krauze et al. [50], who studied the dietary effects of probiotic *Bacillus subtilis* (0.25 g/L) *Enterococcus faecium* (0.25 g/L), and phytobiotics containing cinnamon oil (0.25 mL/L) in broiler chickens and found improvements in the immune system and parameters such as RBCs and Hb. In another experiment, Gilani et al. [11] examined the efficacy of organic acids and phytobiotics (possessing flavonoids) in poultry feed as alternatives to AGPs, observing significant increases in RBC and WBC counts, as well as an increase in PCV in broiler chickens. Similarly, broiler chickens fed Garden cress (*Lepidium satvium*) seed powder [51], cayenne pepper (*Capsicum frutescens*) and turmeric (*Curcuma longa*) powders [52], and pawpaw leaf and seed meal [53] showed increased values of Hb, PCV, and RBCs.

The present study results were not significant (*p* > 0.05) for MCV, MCH, or MCHC in experimental broiler chickens. These results affirm the findings of Oghenebrorhie and Oghenesuvwe, [54], who reported no significant results for MCV, MCH, or MCHC among broilers supplemented with *Moringa oleifera* leaf meal (MOLM)**.**

In conclusion, the dietary supplementation of POLM improved the RBCs, WBCs, PCV, and Hb, suggesting better utilisation of the dietary nutrients.

### 4.3. Serum Biochemistry

Serum biochemical parameters show the metabolism of nutrients in the body and highlight the possible changes resulting from intrinsic and extrinsic factors [55,56]. The liver is one of the largest and most vital organs of living organisms, and it has a pivotal role in detoxification, metabolism, and elimination of endogenous and exogenous substances [57]. The activity levels of ALP, AST, and ALT are considered as diagnostic tools that may be used to evaluate hepatotoxicity [58]. Any pathological manifestation or toxicity results in enhanced activity levels of AST and ALT [59]. Moreover, their activity levels are considered as specific indicators of liver injury or impairment [60]. The current study results showed decreased (*p* < 0.05) serum activity of AST and ALT by increasing the POLM supplementation dosage. However, the serum activity of ALP was not influenced by (*p* > 0.05) POLM supplementation in experimental broiler chickens. The decreased activity of ALT and AST indicated the hepatoprotective nature of the POLM. The POLM possesses a significant concentration of flavonoids and secondary metabolites, including quercetin, which is believed to be responsible for hepatoprotective activity [17,61]. Farag and El-Rayes [62] revealed the hepatoprotective effect of quercetin from bee pollen in broilers, which has an ability to restrict oxidative damage to the liver. Another study by Odetola et al. [12] showed that the graded supplementation of *Petiveria alliacea* root meal in broiler chickens significantly decreased the activity of AST. In a previous study, Oloruntola et al. [63] found that the dietary inclusion of pawpaw and bamboo leaf meal significantly decreased the activity of ALT in broiler chickens.

Serum proteins are primarily synthesised in the liver and their concentrations reflect the functional status of hepatocytes. Any decline in the levels of serum proteins (TP, albumin, and globulin) may be the result of hepatic insufficiency, malnutrition, and active inflammation, which may be due to the recurrent infections and immune deficiency [64]. Furthermore, the serum protein levels of birds are considered important indicators for the determination of their health status. The fattening period of broiler chickens is very short, and there is a rapid accumulation of building proteins in the body tissues, which may significantly influence the concentrations of proteins in the blood, as well as their composition [65]. This rapid growth trend requires intensive erythropoiesis and haemoglobin synthesis, which can result in increased globulin production, potentially affecting the concentrations of serum protein levels in growing chickens [66,67]. The current study results showed that the inclusion of POLM significantly increased the levels of TP, albumin, and globulin compared to the control group. In addition, TP, albumin, and globulin showed linear increases with increasing supplementation of POLM. The present study results are in accordance with the results of Goerge et al. [68], who noted higher serum TP levels in broilers fed a ginger-powder-supplemented diet at starting and finishing phases. Abudabos et al. [69] reported trends for serum TP and globulin for broilers fed anise and thyme essential oils that were in agreement with the current study. 

In birds, the normal reference range of serum glucose is 200 to 500 mg/dL [48]. The present study showed that the serum glucose concentrations were not influenced by POLM in the experimental chickens; however, numerically high values were recorded in the control group compared to supplemented groups. The current findings are in line with the study by Abudabos et al. [69], where serum glucose did not differ significantly in experimental broilers supplemented with phytogenic feed additives.

The serum concentrations of cholesterol and triglycerides are considered to be indicators of lipid metabolism [70]. The current study findings showed that the serum levels of triglycerides and cholesterol were not affected by POLM supplementation at day 21, however significant decreases in the levels of triglyceride and cholesterol were noted in POLM-supplemented groups relative to control on day 42. Furthermore, it was observed that the increasing dosages of POLM linearly decreased the serum levels of triglycerides and cholesterol. The current study results are endorsed by Vispute et al. [13], who reported that dill and hemp seed (possessing flavonoids) significantly decreased the serum levels of triglycerides in the final growth phase. Similarly, our results are in agreement with Zhang et al. [71], who specified that the supplementation of *Chinese bayberry* leaves in chickens’ diets significantly decreased the serum concentrations of triglycerides and cholesterol. Our outcomes are affirmed by Zhou et al. [72] and Niu et al. [73], who reported that dietary supplementation of broilers with fermented *Ginkgo biloba* rations and fermented *Ginkgo biloba* leaves can significantly decrease the serum levels of triglycerides and cholesterol. Similar results were shared by Gilani et al. [11], who revealed that phytobiotics, organic acids, and their combinations resulted in significantly reduced serum levels of cholesterol and triglycerides in broiler chickens. 

Electrolyte balance plays an important role in acid–base balance and ultimately modifies the performance of broiler birds. Any alteration in the acid–base balance results in malfunction of the biochemical and metabolic pathways, which results in an inability to maintain the physiological status of the birds. The minerals Na, K, and Cl are essential for acid–base and osmotic balance, as well as transport of substances across the cell membranes. Thus, they play vital roles in the metabolisms of living organisms. Any imbalance in these minerals can directly alter the acid–base balance, metabolic functions, and ultimately the performance of broiler chickens [74]. In the present study, the Na, K, and Cl values were within normal ranges. These results are in accordance with Malahubban and Ab Aziz, [75], who reported the graded supplementation of Misai Kucing (*Orthosiphon stamineus*) in broiler chicken. 

The kidneys are considered the second target organs that may be injured due to metabolic dysfunctions. Kidney function plays a key role in measuring the possible toxicity of any compound. The status of kidney function can be measured via the increase or decrease in serum levels of urea and creatinine. Higher creatinine levels result from reduced glomerular filtration, which reflects kidney impairment [76], while an elevated serum urea level indicates cardiac and renal tissue injuries. The current study findings showed that serum levels of creatinine and urea were significantly decreased as the POLM dosage increased. These findings indicated that POLM had no deleterious effects on kidney function. Various studies using phytobiotics supplementation in broiler chickens have supported our present study results, including the work by Rubio et al. [77], Ahmad et al. [78], and Adegoke et al. [52].

In conclusion, our findings showed decreased activity of AST and ALT with reduced serum levels of urea and creatinine. These results highlighted that POLM supplementation was useful in terms of liver and kidney function, and was safe even at 8 g/kg in broiler chickens. Additionally, the increased blood protein levels (TP, albumin, and globulin) in this study might be due to the antioxidant and immunomodulatory properties of the POLM supplementation [5,6].

### 4.4. Relative Internal Organs Weights

The relative internal organs weights served as an indicator for the responses of animals towards any in-feed toxic substance that may result in an increase or decrease in internal organs weights [79]. In the current study, no macroscopic alterations, such as hypertrophy or atrophy, injury, and swelling, were noticed in any internal organ. Furthermore, dietary inclusion of POLM did not influence the relative organs weights in experimental broiler chickens. The outcomes of our present study are in agreement with Oloruntola [53], who observed that the relative internal organs weights of the broilers were not influenced by dietary inclusion of seed meal and pawpaw leaf meal. Similar observations were described in the work by Rubio et al. [77] and Vispute et al. [13], where dietary addition of phytobiotics did not influence the relative organs weights in broiler chickens. 

In conclusion, the constant relative internal organs weights of the broilers across experimental groups suggested that, POLM supplementation had no adverse effect on internal organs of the broiler chickens.

### 4.5. Histomorphological Analysis of the Liver

The histomorphological study of the liver revealed that POLM supplementation did not show deleterious effects on liver tissues. The vacuolar degeneration was more frequent in the control group compared to the hepatic tissue samples of all the POLM-supplemented groups. The microscopic characteristics of the hepatic tissues showed positive impacts on the histomorphologies of the livers, as seen in Figure 1b–d compared to Figure 1a (control group). The histomorphological changes in the present study were comparable to the previous study by Quereshi et al. [80], in which the hepatoprotective effects of *fenugreek* seeds and *dandelion* leaves in broiler chickens resulted in normal architecture of the hepatic parenchyma. Additionally, the hepatoprotective effects of these phytobiotics were suggested to be due to the presence of flavonoids in *dandelion* leaves and *fenugreek* seeds. Klaric et al.’s [15] results also supported the results of the present study, where supplementation of phytobiotics such as propolis and bee pollen, which possess flavonoids, ameliorated the liver morphology compared to the control group. Additionally, normal hepatocytes without regressive lesions were noticed in the supplemented groups compared to the control. The control group showed extensive regressive lesions in liver tissue sections. In another study, Farag and El-Rayes [62] observed that the dietary supplementation of bee pollen in broiler chickens’ diets ameliorated the hepatic parenchyma and reduced tissue injury. Furthermore, flavonoids such as quercetin might have a protective effect against oxidative damage of the liver.

Inflammatory responses and oxidative stress are key factors that can damage the liver. Any substance that can diminish the oxidative stress and inflammation can produce hepatoprotective effects and reduce hepatic injury. In the present study, the POLM supplementation produced protective effects on the hepatocytes. These hepatoprotective effects were primarily due to quercetin, which produce effects by limiting oxidative stress [41]; pro-inflammatory cytokines such as TNF-α, IL-6, and COX-2 [42]; and nuclear factor NF-κB, probably via interference of the signalling of the toll-like receptor TLR_4_ [81]. Furthermore, quercetin increases the non-enzymatic and enzymatic antioxidants by stimulating the Nrf2–ARE signalling pathway in cells, which might positively influence the liver status and function [82]. Conclusively, increasing the POLM supplementation levels has a gradual ameliorating histomorphological effect on hepatocytes. A predominantly healthy architecture of the liver parenchyma was noticed in POLM-supplemented group Po8 compared to the Po2 and control groups.

## 5. Conclusions

The current study results showed that the dietary inclusion of POLM supplementation in broiler chickens enhanced the growth performance and positively improved haematological blood indicators and serum biochemistry attributes, with no deleterious effects on the internal organs. Additionally, broilers chicken fed a diet supplemented with POLM at a rate of 8 g/kg showed the most promising results in terms of growth performance, as well as for the tested blood and serum biochemistry parameters, and retained relatively normal hepatic parenchyma. Thus, POLM supplementation at 8 g/kg would be the appropriate dose as an alternative feed additive for broiler chickens.

## Figures and Tables

**Table 1:** Ingredients (% as feed) and nutritional analysis of the basal diet.

Ingredients %	Starter Period	Finisher Period
Corn	54.40	58.95
Soybean Meal (SBM) (44%)	33.90	28.00
Fish Meal	5.33	5.74
Palm Oil	2.64	4.14
Salt	0.38	0.28
Limestone	1.06	0.86
Dicalcium Phosphate	1.09	0.84
Mineral Mix ^††^	0.28	0.28
Vitamin Mix ^†^	0.29	0.29
DL-Methionine	0.18	0.18
Choline chloride	0.10	0.10
L-Lysine	0.35	0.34
Calculated analysis (%) *		
Crude Protein, %	22.00	20.00
Metabolise Energy (ME) MJ/kg	13.10	13.40
Crude Fat, %	5.21	7.01
Available P, %	0.43	0.35
Calcium, %	0.99	0.90

^†^ Premixed administered vitamins per (kg) of dietary feed: Vitamin K (menadione) 1.33 (mg); Vitamin A (retinol), 1950 (μg); Vitamin D_3_ 30 (μg); Vitamin E, 0.02 (mg); riboflavin, 2.0 (mg); Biotin, 0.03 (mg); Vitamin B_12_, 0.03 (mg); VitaminB_1_, 0.83 (mg); Vitamin B_3_ 24 (mg); Vitamin B_6_, 1.37 (mg); Folic acid, 0.33 (mg); Calcium D-Panthothenate, 3.69 (mg). ^††^ Premixed administered minerals per kg of dietary feed: Zinc, 100.01 (mg); iron, 120.0 (mg); Mg, 16.0 (mg); I, 0.8 (mg); Co, 0.6 (mg); Cu, 19.99 (mg). Diet C = Control (0 g/kg medicinal herb; *P. odorata*); Diet Po2 = 2 g/kg *P. odorata*; Diet Po4 = 4 g/kg *P. odorata*; Diet Po8 = 8 g/kg *P. odorata*; * Calculated according to NRC [28].

**Table 2:** Effects of different doses of supplementation of *Persicaria odorata* leaf meal (POLM) on the growth performance of broiler chickens.

Parameters	Treatment					*p*-Value	*p*-Value of Contrast	
	C	PO2	PO4	PO8	SEM	ANOVA	Linear	Quadratic
**Initial BWT (g/bird)**	40.31	40.30	40.32	40.30	0.031	0.994	0.9901	0.956
**FI (g/d/bird)**								
1–21 d	48.69	49.30	49.11	49.47	0.361	0.942	0.975	0.924
22–42 d	142.95	144.10	143.97	143.51	0.412	0.788	0.802	0.836
1–42 d	95.82	96.70	96.54	96.49	0.287	0.749	0.943	0.995
**BWG (g/d/bird)**								
1–21 d	30.04 ^b^	31.38 ^a,b^	31.94 ^a,b^	32.97 ^a^	0.372	0.048	0.164	0.974
22–42 d	78.06 ^b^	80.50 ^a^	80.68 ^a^	82.53 ^a^	0.499	0.005	0.047	0.725
1–42 d	54.23 ^c^	55.94 ^b^	56.31 ^b^	57.65 ^a^	0.340	0.0003	0.003	0.430
**FCR**								
1–21 d	1.60	1.57	1.54	1.51	0.021	0.215	0.307	0.999
22–42 d	1.83 ^a^	1.79 ^a,b^	1.78 ^a,b^	1.74 ^b^	0.012	0.048	0.047	0.908
1–42 d	1.77 ^a^	1.73 ^a^	1.72 ^ab^	1.67 ^b^	0.011	0.012	0.004	0.549
**Mortality rate (%) 1–42 d**								
	6.66	0.00	3.66	0.00	-	-	-	-

^a–c^ indicate that values in the same row with different superscripts are significantly different (*p* < 0.05). FI: feed intake; BWG: body weight gain; FCR: feed conversion ratio; C: control; basal diet alone; Po2: basal diet+ POLM 2 g/kg; Po4: basal diet+ POLM 4 g/kg; Po8: basal diet+ POLM 8 g/kg. SEM: standard error of mean.

**Table 3:** Haematological blood indicators of broilers fed with different supplementation doses of POLM at day 21.

Parameters	Treatment					*p*-Value	*p*-Value of Contrast	
	C	PO2	PO4	PO8	SEM	ANOVA	Linear	Quadratic
RBCs (mm^3^ × 10^6^)	2.39 ^b^	2.70 ^a,b^	2.78 ^a,b^	2.92 ^a^	0.08	0.036	0.048	0.545
PCV (%)	30.38 ^b^	32.50 ^a^	33.13 ^a^	32.86 ^a^	0.37	0.006	0.218	0.950
Hb (g/dL)	9.35 ^b^	10.55 ^a,b^	10.84 ^a^	10.94 ^a^	0.25	0.035	0.048	0.999
MCV (fL)	129.72	122.77	126.43	115.12	2.76	0.219	0.088	0.234
MCH (pg)	39.73	39.26	39.40	38.04	0.56	0.586	0.813	0.158
MCHC (%)	30.85	32.39	34.26	34.79	0.57	0.535	0.151	0.974
WBCs (mm^3^ × 10^6^)	22.49 ^b^	22.66 ^b^	23.84 ^a,b^	24.23 ^a^	0.27	0.032	0.0001	0.430

^a,b^ indicate that values in the same row with different superscripts are significantly different (*p* < 0.05). RBCs: red blood cells; PCV: packed cell volume; Hb: haemoglobin; MCV: mean corpuscular volume; MCH: mean corpuscular haemoglobin; MCHC: mean corpuscular haemoglobin concentration; WBCs: white blood cells; C: control; basal diet alone; Po2: basal diet+ POLM 2 g/kg; Po4: basal diet+ POLM 4 g/kg; Po8: basal diet+ POLM 8 g/kg.

**Table 4:** Haematological blood indicators of broilers fed on different supplementation doses of POLM at day 42.

Parameters	Treatment					*p*-Value	*p*-Value of Contrast	
	C	PO2	PO4	PO8	SEM	ANOVA	Linear	Quadratic
RBCs (mm^3^ × 10^6^)	2.54 ^b^	2.85 ^a,b^	2.89 ^a,b^	3.00 ^a^	0.07	0.069	0.046	0.800
PCV (%)	31.14 ^b^	33.34 ^a,b^	33.40 ^a,b^	33.89 ^a^	0.40	0.039	0.231	0.559
Hb (g/dL)	9.62 ^b^	10.50 ^a,b^	10.82 ^a,b^	11.13 ^a^	0.22	0.047	0.047	0.980
MCV (fL)	123.43	120.03	117.21	113.76	2.05	0.215	0.016	0.990
MCH (pg)	38.02	37.66	37.75	37.15	0.46	0.671	0.205	0.641
MCHC (%)	30.86	31.59	32.38	32.75	0.35	0.202	0.039	0.710
WBCs (mm^3^ × 10^6^)	22.67 ^b^	22.86 ^b^	23.98 ^a,b^	25.04 ^a^	0.33	0.027	0.0001	0.998

^a,b^ indicate that values in the same row with different superscripts are significantly different (*p* < 0.05). RBCs: red blood cells; PCV: packed cell volume; Hb: haemoglobin; MCV: mean corpuscular volume; MCH: mean corpuscular haemoglobin; MCHC: mean corpuscular haemoglobin concentration; WBCs: white blood cells; C: control; basal diet alone; Po2: basal diet+ POLM 2 g/kg; Po4: basal diet+ POLM 4 g/kg; Po8: basal diet+ POLM 8 g/kg.

**Table 5:** Biochemical indicators of chicken blood from birds fed different supplementation doses of POLM at day 21.

Parameters	Treatment					*p*-Value	*p*-Value of Contrast	
	C	PO2	PO4	PO8	SEM	ANNOVA	Linear	Quadratic
ALP (U/L)	1794.1	1801.00	1819.20	1863.10	16.94	0.487	0.048	0.828
AST (U/L)	216.8 ^a^	196.9 ^b^	190.1 ^c^	183.7 ^d^	2.18	<0.0001	0.000	0.985
ALT (U/L)	6.84 ^a^	7.02 ^a^	6.97 ^a^	6.36 ^b^	0.06	<0.0001	0.000	0.000
Total protein (g/dL)	2.311 ^c^	2.430 ^b,c^	2.540 ^a,b^	2.650 ^a^	0.03	0.001	0.000	1.000
Albumin (g/dL)	1.112 ^b^	1.212 ^a^	1.229 ^a^	1.287 ^a^	0.02	0.015	0.022	0.662
Globulin (g/dL)	1.198 ^b^	1.218 ^ab^	1.311 ^a^	1.364 ^a^	0.02	0.040	0.000	0.769
Glucose (mmol/L)	14.90	14.30	13.70	13.10	0.58	0.744	0.380	1.000
Cholesterol (mmol/L)	3.00	2.98	2.97	2.82	0.04	0.477	0.056	0.473
Triglycerides (mmol/L)	0.93	0.92	0.93	0.91	0.01	0.898	0.727	0.636
Na (mmol/L)	128.5^b^	132.90 ^a,b^	136 ^a,b^	137.9 ^a^	1.48	0.049	0.060	0.943
K (mmol/L)	5.23	4.35	4.18	4.09	0.19	0.145	0.627	0.985
Cl (mmol/L)	106.4	108.66	108.9	109.6	1.55	0.904	0.918	0.993
Urea (mmol/L)	0.44 ^a^	0.40 ^b^	0.38 ^c^	0.37 ^c^	0.01	<0.0001	0.044	0.924
Creatinine (mmol/L)	28.28 ^a^	28.65 ^a^	24.48 ^b^	23.45 ^b^	0.68	0.005	0.000	0.112

^a–d^ indicate that values in the same row with different superscripts are significantly different (*p* < 0.05). ALP: alkaline phosphatase; AST: aspartate aminotransferase; ALT: alanine aminotransferase; C: control; basal diet alone; Po2: basal diet+ POLM 2 g/kg; Po4: basal diet+ POLM 4 g/kg; Po8: basal diet+ POLM 8 g/kg.

**Table 6:** Biochemical indicators of chicken blood from birds fed different supplementation doses of POLM at day 42.

Parameters	Treatment					*p*-Value	*p*-Value of Contrast	
	C	PO2	PO4	PO8	SEM	ANNOVA	Linear	Quadratic
ALP (U/L)	1650.19	1659.70	1652.80	1671.80	13.27	0.943	0.825	0.722
AST (U/L)	224.50 ^a^	203.70 ^b^	200.00 ^b^	195.80 ^b^	2.27	<0.0001	0.003	0.991
ALT (U/L)	8.34 ^a^	6.98 ^b^	6.95 ^b^	5.75 ^c^	0.19	<0.0001	0.000	0.004
Total protein (g/dL)	2.418 ^c^	2.582 ^b^	2.668 ^b^	2.744 ^a^	0.03	0.0008	0.001	0.988
Albumin (g/dL)	1.180 ^b^	1.269 ^a,b^	1.251 ^a,b^	1.311 ^a^	0.02	0.049	0.313	0.279
Globulin (g/dL)	1.238 ^b^	1.313 ^a,b^	1.417 ^a^	1.434 ^a^	0.03	0.027	0.006	0.374
Glucose (mmol/L)	15.90	15.70	14.20	13.30	0.63	0.414	0.021	0.874
Cholesterol (mmol/L)	3.23 ^a^	3.03 ^b^	2.95 ^b^	2.77 ^c^	0.04	<0.0001	0.000	0.451
Triglycerides (mmol/L)	1.08 ^a^	0.94 ^b^	0.80 ^c^	0.75 ^c^	0.03	<0.0001	0.000	0.355
Na (mmol/L)	136.1	136.60	140.10	141.6	1.42	0.461	0.059	0.850
K (mmol/L)	5.55 ^a^	4.53 ^b^	4.24 ^b^	4.03 ^b^	0.19	0.020	0.149	0.985
Cl (mmol/L)	112.3	109.93	110	109.3	1.68	0.933	0.968	0.984
Urea (mmol/L)	0.48 ^a^	0.42 ^b^	0.36 ^c^	0.35 ^c^	0.01	<0.0001	0.000	0.031
Creatinine (mmol/L)	31.11 ^a^	30.07 ^a^	23.73 ^b^	21.14 ^b^	0.89	<0.0001	0.000	0.022

^a–c^ indicate that values in the same row with different superscripts are significantly different (*p* < 0.05). ALP: alkaline phosphatase; AST: aspartate aminotransferase; ALT: alanine aminotransferase; C: control; basal diet alone; Po2: basal diet+ POLM 2 g/kg; Po4: basal diet+ POLM 4 g/kg; Po8: basal diet+ POLM 8 g/kg.

**Table 7:** Dressing percentages and relative internal organs weights of broilers fed different supplementation doses of POLM at day 42.

Parameters	Treatment					*p*-Value	*p*-Value of Contrast	
	C	PO2	PO4	PO8	SEM	ANNOVA	Linear	Quadratic
Dressing %	71.16	71.85	73.09	74.35	0.60	0.367	0.040	1.000
Gizzard	1.88	2.18	2.2	2.28	0.09	0.218	0.897	0.987
Liver	2.31	2.27	2.25	2.31	0.09	0.978	0.888	0.863
Heart	0.63	0.61	0.6	0.61	0.04	0.973	0.990	0.964
Kidney	0.61	0.59	0.63	0.62	0.03	0.950	0.683	0.737
Spleen	0.13	0.13	0.14	0.15	0.01	0.146	0.000	0.440
Pancreas	0.21	0.23	0.23	0.23	0.01	0.894	0.920	0.979
Bursa of Fabricius	0.2	0.22	0.21	0.22	0.01	0.675	0.949	0.222

C: control; basal diet alone; Po2: basal diet+ POLM 2 g/kg; Po4: basal diet+ POLM 4 g/kg; Po8: basal diet+ POLM 8 g/kg.
